# Alterations in sleep-activity cycles and clock gene expression across the synucleinopathy spectrum

**DOI:** 10.1186/s40035-025-00492-7

**Published:** 2025-06-03

**Authors:** Maria Comas, Xavier Vidal, Oliver Rawashdeh, Ronald R. Grunstein, Simon J. G. Lewis, Elie Matar

**Affiliations:** 1 Centre for Integrated Research and Understanding of Sleep (CIRUS), Woolcock Institute of Medical Research, 2 Innovation Rd, Macquarie Park, NSW 2113 Australia; 2 TECNALIA, Basque Research and Technology Alliance (BRTA), Parque Científico y Tecnológico de Bizkaia, Astondo bidea, 700, 48160 Derio, Spain; 3 School of Biomedical Sciences, Faculty of Medicine, The University of Queensland, Brisbane, QLD 4072 Australia; 4 Macquarie Medical School and Macquarie University Centre for Parkinson’s Disease Research, Faculty of Medicine, Health and Human Sciences, Macquarie University, 75 Talavera Rd, Macquarie Park, Sydney, NSW 2113 Australia; 5 Department of Neurology, Royal Prince Alfred Hospital, Camperdown, NSW 2006 Australia; 6 Parkinson’s Disease Research Centre, Brain and Mind Centre, Central Clinical School, Faculty of Medicine and Health, The University of Sydney, 100 Mallet Street, Camperdown, NSW 2050 Australia

Sleep disturbances are common in synucleinopathies such as Parkinson's disease (PD) and dementia with Lewy bodies (DLB) suggesting a central role in their pathophysiology and progression. Isolated REM sleep behavior disorder (iRBD), a parasomnia, frequently precedes PD and DLB and is now regarded as a prodromal synucleinopathy [1]. The biological clock, regulated by clock genes and secretion of the hormone melatonin, coordinates various systems including sleep, metabolism, immune and endocrine function [2]. Dysregulation of melatonin levels and clock gene expression rhythms has been reported in iRBD and PD [3, 4], with mixed findings, and has not yet been studied in DLB. There is a need for clinical and biological markers that predict the risk of phenoconversion in patients with iRBD, and their trajectory towards either DLB or PD.

We investigated circadian function in patients with early but established PD and DLB, a prodromal iRBD group and age-matched healthy controls (HC). We hypothesized that disruption in daily rhythms would occur in a graded manner across the continuum of synucleinopathies, with increasing severity from HC to iRBD, then PD and DLB, reflecting the perceived severity of pathology. To assess this hypothesized monotonic trend in sleep and circadian disruption across the groups, we used the Jonckheere-Terpstra test which is appropriate for detecting ordered differences in non-parametric data. Disease group was used as the independent variable allowing us to test whether circadian measures progressively worsen along the continuum of synucleinopathies (DLB > PD > iRBD > HC). For rhythmicity assessments, we instead applied harmonic regression models. Detailed methods are provided in Additional file 1.

The demographic, clinical, as well as subjective sleep and circadian measures of the participants are summarized in Table S1. Age did not differ between DLB and controls. Chronotype scores indicated moderate morning preference in all groups except iRBD, which showed an intermediate profile. The Epworth Sleepiness Scale (ESS) demonstrated lower normal daytime sleepiness (scores 0–5) in the iRBD group (5.6 ± 4.5), higher normal daytime sleepiness (scores 6–10) for HC (6.7 ± 3.1) and PD (7.25 ± 3.7), and mild excessive daytime sleepiness (scores 11–12) for the DLB group (11.8 ± 5.2), which was significantly higher than all other groups. Subjective night-time sleep disturbance measured by Scales for Outcomes in PD-Sleep Scale (SCOPA-S) (cutoff for poor sleep quality > 6) showed values below cutoff in all groups with no significant difference. Subjective day-time sleep disturbance measured by SCOPA-S (cutoff for poor sleep quality > 4) showed values below the cutoff for all groups except the DLB group (8.9 ± 4.9), which reported significantly worse daytime somnolence compared to all other groups. We found a significant pattern of increase of the median day-time sleep disturbance measured by SCOPA-S from controls to PD and DLB (two-sided, *T*_JT_ = 923.00, *z* = 4.114, *P* < 0.001). A similar pattern of increase of daytime sleepiness measured by the ESS was seen across groups (two-sided, T_JT_ = 1122.00, *z* = 3.207, *P* = 0.001).

The averaged rest-activity profiles measured by actigraphy (Actiwatch 2, Philips Healthcare) showed that all four groups had significant activity rhythm profiles (Fig. 1a). A cosinor analysis was performed to derive the activity mesor, amplitude, and acrophase (Table S2; Fig. 1a). The test for ordered alternatives showed that there was a significant pattern of decreasing median mesor of the rest-activity rhythm from controls, to iRBD, to PD and then to DLB (two-sided, *T*_JT_ = 225.00, *P* < 0.001). Similarly, a significant trend was found for the amplitude of the rest-activity rhythm, being significantly higher in the HC (87.4 ± 13) group and lowest for the DLB (47.2 ± 27.3) group (two sided, *T*_JT_ = 205.00, *P* < 0.001). A *post-hoc* partial correlation assessing the relationship between rest-activity mesor and motor parkinsonism controlling for diagnosis and disease duration revealed a statistically significant, moderate-strength, negative correlation between UPDRS-III score and activity mesor (*r*(33) = −0.38, *P* = 0.024) as well as amplitude (*r*(33) = −0.46, *P* = 0.006). No significant trend was seen for the acrophase of the rest-activity rhythm across groups. Sleep measures derived from actigraphy are summarised in Table S3. Bedtime, rise time, time in bed, total sleep time, and 24-h sleep duration did not differ significantly. Wake time after sleep onset was significantly shorter in the PD group compared to DLB (*F* = 5.273, *P* = 0.004). Sleep fragmentation was lower in the HC and PD groups compared to DLB (*F* = 6.008, *P* = 0.002). The activity counts over 24 h were significantly higher in the HC group compared to DLB (*F* = 4.478, *P* = 0.008). Activity counts during the night were lower in the iRBD and PD compared to DLB (*F* = 3.739, *P* = 0.018). Percentage of wake during the night was lower in the PD compared to DLB (*F* = 5.777, *P* = 0.002).

**Fig. 1:**
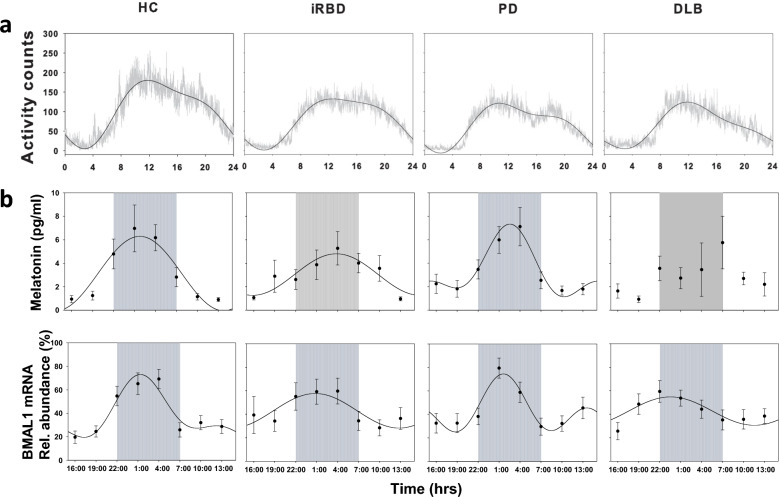
Averaged actigraphy profiles, as well as melatonin and *Bmal1* expression levels of HC, iRBD, PD and DLB patients. **a** Activity was recorded during 7 days and the averaged activity levels per participant and group is shown in a solid grey line in intervals of 30 s. Black solid lines indicate harmonic regression fits (fundamental wave and first harmonic). **b** Salivary melatonin levels determined by radioimmunoassay and *Bmal1* expression by quantitative PCR using oral mucosa samples. Salivary and oral mucosa samples were collected every three hours during the 24-h cycle. Values are expressed as means (black circles) ± SEM.. Solid black lines indicate harmonic regressions

Salivary melatonin secretion sampled every 3 h showed that across HC, iRBD and PD, one fundamental sine wave contributed significantly to the explained variance (Fig. 1b). No harmonics could be fitted for the melatonin data obtained from the DLB group, meaning that there was no circadian variation of melatonin in these patients.

The *Bmal1* expression profiles determined by qPCR using oral mucosa samples showed that a fundamental sine wave was significantly fitted to the *Bmal1* gene expression profile across all groups (Fig. 1b). There was a significant pattern of decrease of median *Bmal1* amplitude from controls, to iRBD, to PD and then to DLB (two sided,* T*_JT_ = 393.00, *P* = 0.037). No statistically significant trend was seen for the *Bmal1* mesor or acrophase across groups.

In summary, we found a significant and progressive sleep/wake cycle disruption along the synucleinopathy spectrum with respect to actigraphy measures of rest-activity profiles and the expression of *Bmal1*. This is the first study to demonstrate disruption of clock gene expression and melatonin in patients with DLB. In our dataset, no significant alterations in melatonin rhythms were found between controls, iRBD, and PD groups. Previous circadian melatonin secretion studies in synucleinopathies have shown mixed results on melatonin rhythms in early-stage PD patients [5, 6]. The differences in melatonin findings in PD may reflect lower severity and short duration of disease in PD, heterogeneity or simply a lack of power to detect a modest effect. However, we found that melatonin secretion in our DLB group did not show a significant oscillation (Fig. 1b), suggesting a significant disruption of the circadian clock, at least for this hormonal output.

Actigraphy could be an objective marker of motor symptom progression in iRBD, as measures of activity/rest rhythms reveal progressively reduced activity in a manner that correlates with motor parkinsonism. Actigraphy could also differentiate between groups, showing significant differences in measures of sleep/rest between groups. Our results were consistent with previous findings of excessive daytime somnolence in DLB [7], which is associated with cholinergic neuronal loss within the nucleus basalis of Meynert [8]. Such measures may therefore be more a marker of pathological progression towards dementia specifically.

Strengths of this study include thorough phenotyping, confirmation of RBD through video polysomnography, and the collection of easily obtainable saliva and oral mucosa samples, enhancing the clinical and research utility of the approach. Limitations include its cross-sectional design, which precludes causal inference, and a short actigraphy monitoring period (7 days). Although our study sample is comparable or greater than those previously published, it is possible that the study was underpowered for some of the measurements.

Medication use may be a confounder. Most PD and approximately half of the DLB participants were receiving dopaminergic therapy. Prior studies report mixed effects of these treatments on melatonin phase and secretion [6, 9]. Their impact on peripheral clock gene expression remains unclear, though one study found reduced *Bmal1* expression in PD independent of medication [10]. Effects on rest-activity rhythms also vary, potentially influenced by age or disease/treatment duration [9]. Acetylcholinesterase inhibitors, used by only four DLB participants, are even less studied. Details are in Table S1. The small sample limited statistical control for all medication effects.

Altogether, our findings show that altered sleep and daily rhythms, including changes in rest-activity cycles and reduced *Bmal1* expression, reflect increased pathological severity across the synucleinopathy spectrum. Thus, circadian disruption may be a potential biomarker for disease progression and phenoconversion in these disorders, offering insights for future therapeutic strategies.

## Supplementary Information

Additional file 1. **Methods**. **Table S1**. Average and standard deviation of demographic, clinical and sleep measures. **Table S2**. Cosinor analysis of actigraphy, melatonin and Bmal1 expression. **Table S3**. Average and standard deviation of actigraphy variables. 

## Data Availability

The datasets used and/or analysed during the current study are available from the corresponding author on reasonable request.

## Abbreviations

DLB: Dementia with Lewy bodies
ESS: Epworth Sleepiness Scale
HC: Healthy control
iRBD: Isolated rapid eye movement sleep behaviour disorder
PD: Parkinson's disease
