# Supplementary material for: Alterations in sleep-activity cycles and clock gene expression across the synucleinopathy spectrum
Source: Transl Neurodegener. 2025 Jun 3;14:28. doi: 10.1186/s40035-025-00492-7 (PMC12131590; doi:10.1186/s40035-025-00492-7)
Supplement: Supplementary file 1 — Additional file 1. Methods. Table S1. Average and standard deviation of demographic, clinical and sleep measures. Table S2. Cosinor analysis of actigraphy, melatonin and Bmal1 expression. Table S3. Average and standard deviation of actigraphy variables. [file 40035_2025_492_MOESM1_ESM.docx]

# Methods

***Participants***

Sixty-eight participants with a diagnosis of PD (*n*=16), DLB (*n*=17), iRBD (*n*=20) or age matched healthy controls (HC) (*n*=15) were recruited from the Parkinson’s Disease Research Clinic at the Brain and Mind Centre, University of Sydney and via community advertisements. All participants underwent a detailed neurological assessment by S.J.G.L. and E.M. at the Brain and Mind Centre, in Sydney (Australia). Video polysomnography (PSG) of all participants was performed at the Woolcock Institute of Medical Research. The presence of RBD was confirmed by clinical diagnosis using video polysomnography and met the diagnostic criteria according to the International Classification of Sleep Disorders-3 (1). All PD patients had been diagnosed within 5 years of the study according to the Movement Disorders Society (MDS) PD diagnostic criteria (2). The DLB patients satisfied the consensus criteria of the Fourth report of the DLB Consortium and were all within 3 years of diagnosis at the time of the study (3). Motor function was assessed using the motor section of the MDS Unified Parkinson’s Disease Rating Scale (MDS-UPDRS-III) (4). Healthy controls underwent the same procedures as patients, including neurological assessment. In our cohort, medication use was carefully documented. No subjects were taking anti-psychotics, beta-blockers or neuroleptic medications at the time of the study. Six subjects (2 RBD, 3 PD and 1 DLB were taking antidepressants). Dopaminergic medications were limited to participants with PD and DLB, with 7 participants with DLB taking dopaminergic medications (namely levodopa/carbidopa) with the dopamine dose equivalence as highlighted in Table S1. Acetylcholinesterase inhibitors (rivastigmine) were limited to 4 participants with DLB. The study was approved by Royal Prince Alfred Hospital Ethics Review Committee, Sydney, Australia Protocol No X15-0207 & HREC/15/RPAH/272. All participants provided written informed consent prior to commencement of the study.

**Procedures**

Participants completed general health and lifestyle questionnaires, as well as neurological and sleep questionnaires detailed in Table 1. Sleep-wake activity was objectively assessed via actigraphy and all participants stayed in the sleep laboratory at the Woolcock Institute of Medical Research for 24 hours, where they underwent overnight PSG following the SINBAR protocol (5). During this time, salivary and oral mucosa samples were collected every three hours (over the 24-hour period) to measure melatonin and *Bmal1* clock gene expression. Illuminance in the room during daytime hours (~7am-10pm) was ~100 lux and at lights-out <1 lux.

***Questionnaires***

A series of questionnaires evaluating mood and sleep were completed as shown in Table 1. Cognitive performance was evaluated with the Montreal Cognitive Assessment (MoCA) (6) and the Mini-Mental State Examination (MMSE) (7). The Hospital Anxiety and Depression Scale (HADS) was used to assess anxiety (HADS-A) and depressive symptoms (HADS-D) (8). The Morningness-Eveningness Questionnaire (MEQ) was used to assess chronotype (9). The Epworth Sleepiness Scale (ESS) was used to derive an estimate of daytime somnolence (10). The Scales for Outcomes in PD-Sleep Scale (SCOPA-S) was used to estimate both night-time sleep (NS) and day-time sleep (DS) quality (11).

***Actigraphy***

Participants wore a Philips Respironics Actiwatch 2 (Koninklijke Philips N.V., Amsterdam, Netherlands) on the non-dominant wrist for at least one week. Manual scoring of actigraphy and analysis of sleep parameters was performed using Philips Respironics Actiware-5 software (Koninklijke Philips N.V.).

***Salivary melatonin***

Melatonin was sampled every three hours for a period of 24 hours. Sampling started at 16:00 and continued every 3 hours until 13:00 on the next day. During night-time hours (10 pm-7 am) saliva sampling occurred in dim light conditions (<10 lux). Samples were stored immediately in a -80 ^0^C freezer until analysis. Salivary melatonin concentrations were determined by radioimmunoassay (Buhlmann Laboratories; Allschwil, Switzerland). These assays have a limit of detection of 1 pg/mL with an inter-assay coefficient of variations of 7.4% at 4.41 pg/mL and 10.7% at 48.14 pg/mL.

***Bmal1 gene expression***

Oral mucosa samples were collected at the same times as saliva samples. They were obtained by ‘scratching’ the inner cheek for one minute with a cytological brush. The samples were immediately placed into RNAlater reagent (Life Technologies Australia Pty Ltd, Mulgrave, VIC, Australia) and stored at -20 ^0^C until analysis. The mRNA was isolated using the Isolate II RNA Micro kit (Bioline, London, UK). SuperScript VILO cDNA Synthesis kit (Thermofisher) was used to reverse-transcribe the mRNA samples. Quantitative PCR was performed using the ABI Step one Plus detection system and data were analyzed with StepOne software (Applied Biosystems, Melbourne, VIC, Australia). Assays were carried out using Taqman Universal MMIX II with UNG and TaqMan Gene Expression assays for *Bmal1* (Hs00154147_m1) and *Gapdh* (Hs99999905_m1) according to protocol of manufacturer (Life Technologies Australia Pty Ltd, Mulgrave, VIC, Australia).

***Data analysis***

The averaged activity levels measured by actigraphy were plotted for each patient group in 30 seconds-bins of the 24-hour interval averaged over 7 days of recording. Significance of the rhythmicity of activity levels, melatonin and *Bmal1* expression was determined with the CircWave v 1.4 software developed by R.A. Hut, University of Groningen, NL (12-14), which uses a linear harmonic regression fit with an assumed period of 24 hours. Cosinor analysis was performed and cosine curve parameters (amplitude, acrophase and mesor) obtained using the toolbox developed by (15) for MATLAB and Statistics Toolbox Release 2012b, The MathWorks, Inc., Natick, Massachusetts, United States. GraphPad Prism 9.0 was used for statistical analysis. Demographic, questionnaire, and actigraphy data were compared by ANOVA. Post hoc analyses were performed using Tukey's honest significant difference post hoc test. Rhythmicity differences between groups (activity-rest rhythms, melatonin and *Bmal1* expression) were assessed using the Jonckheere-Terpstra test for ordered alternatives, in line with the *a priori* hypothesis that circadian abnormalities would differ in accordance with expected degree of neuropathological disease - with controls being least affected, followed by iRBD, PD and then DLB (most affected). Post-hoc partial correlations were performed using SPSS (version 26.0.0, IBM) with controlling variables as specified. Results are shown as mean ± standard deviation or median (interquartile range) as specified. Values of P < 0.05 were considered statistically significant.

**References**

1. Videnovic A. American Academy of Sleep Medicine. International classification of sleep disorders, 3rd edn.2014.

2. Postuma RB, Berg D, Stern M, Poewe W, Olanow CW, Oertel W, et al. MDS clinical diagnostic criteria for Parkinson's disease. Movement Disorders. 2015;30(12):1591-601.

3. McKeith IG, Boeve BF, Dickson DW, Halliday G, Taylor J-P, Weintraub D, et al. Diagnosis and management of dementia with Lewy bodies. Fourth consensus report of the DLB Consortium. 2017;89(1):88-100.

4. Goetz CG, Fahn S, Martinez-Martin P, Poewe W, Sampaio C, Stebbins GT, et al. Movement Disorder Society-sponsored revision of the Unified Parkinson's Disease Rating Scale (MDS-UPDRS): Process, format, and clinimetric testing plan. Movement Disorders. 2007;22(1):41-7.

5. Frauscher B, Iranzo A, Högl B, Casanova-Molla J, Salamero M, Gschliesser V, et al. Quantification of Electromyographic Activity During REM Sleep in Multiple Muscles in REM Sleep Behavior Disorder. Sleep. 2008;31(5):724-31.

6. Nasreddine ZS, Phillips NA, Bédirian V, Charbonneau S, Whitehead V, Collin I, et al. The Montreal Cognitive Assessment, MoCA: A Brief Screening Tool For Mild Cognitive Impairment. Journal of the American Geriatrics Society. 2005;53(4):695-9.

7. Folstein MF, Folstein SE, McHugh PR. “Mini-mental state”: A practical method for grading the cognitive state of patients for the clinician. Journal of Psychiatric Research. 1975;12(3):189-98.

8. Zigmond AS, Snaith RP. The Hospital Anxiety and Depression Scale. Acta Psychiatrica Scandinavica. 1983;67(6):361-70.

9. Horne JA, Ostberg O. A self-assessment questionnaire to determine morningness-eveningness in human circadian rhythms. International Journal of Chronobiology. 1976;4(2):97–110.

10. Johns MW. A New Method for Measuring Daytime Sleepiness: The Epworth Sleepiness Scale. Sleep. 1991;14(6):540-5.

11. Marinus J, Visser M, van Hilten JJ, Lammers GJ, Stiggelbout AM. Assessment of Sleep and Sleepiness in Parkinson Disease. Sleep. 2003;26(8):1049-54.

12. Comas M, Beersma DGM, Hut RA, Daan S. Circadian Phase Resetting in Response to Light-Dark and Dark-Light Transitions. Journal of Biological Rhythms. 2008;23(5):425-34.

13. Lincoln GA, Clarke IJ, Hut RA, Hazlerigg DG. Characterizing a Mammalian Circannual Pacemaker. Science. 2006;314(5807):1941-4.

14. Oster H, Damerow S, Hut RA, Eichele G. Transcriptional profiling in the adrenal gland reveals circadian regulation of hormone biosynthesis genes and nucleosome assembly genes. J Biol Rhythms. 2006;21(5):350-61.

15. Cheart. cheart (2020). Cosinor Analysis (https://www.mathworks.com/matlabcentral/fileexchange/20329-cosinor-analysis), MATLAB Central File Exchange. Retrieved May 25, 2020. 2020.

| Variable | HC *n*=15  (6 Females) | iRBD *n*=20  (4 Females) | PD *n*=16  (6 Females) | DLB *n*=17  (2 Females) | ANOVA F  *P* value |
| --- | --- | --- | --- | --- | --- |
| Age | 67.3 (9.5) | 66.6 (7.3) ^bd*^ | 63.7 (10.5)^cd**^ | 74.3 (6.7) | **4.631**  **0.0054** |
| Disease duration (years) | - | 7.9 (7.2) ^bc**, bd***^ | 2.7 (1.8) | 1.3 (1.1) | **10.45**  **0.0002** |
| Years of education | 13.1 (2.5) | 14.6 (3.1)^bd*^ | 14.7 (3.4)^cd*^ | 11.5 (3.4) | **4.034**  **0.0109** |
| MoCA | 27.3 (2.35) ^ad***^ | 27.9 (1.6)^bd***^ | 28.8 (1.3)^cd***^ | 16.9 (6.6) | **37.91**  **<0.0001** |
| MMSE | 29.2 (0.9) ^ad***^ | 29.05 (1.02) ^bd***^ | 29.3 (1.3) ^cd***^ | 20.6 (6.4) | **27.12**  **<0.0001** |
| HADS-A | 2.9 (2.2) ^ad*^ | 3.6 (2.6) ^bd*^ | 3.9 (3.2) | 6.5 (4.2) | **3.899**  **0.0129** |
| HADS-D | 1.8 (2) ^ad***^ | 2.5 (3.7) ^bd***^ | 3.4 (3) ^cd***^ | 8.7 (5.1) | **11.32**  **<0.0001** |
| HADS-Total | 4.7 (3.9) ^ad***^ | 6.1 (5.7) ^bd***^ | 7.3 (5.9) ^cd**^ | 15.2 (8.1) | **9.206**  **<0.0001** |
| MEQ | 60.9 (6.3) | 58.1 (7.7) | 59.5 (9.1) | 62.8 (9.2) | 1.089  0.3604 |
| ESS | 6.7 (3.1) ^ad**^ | 5.6 (4.5) ^cd*^ | 7.25 (3.7) ^bd***^ | 11.8 (5.2) | **7.046**  **0.0004** |
| SCOPA-Sleep NS | 4.3 (2.3) | 2.9 (2.5) | 3.6 (3.1) | 4.5 (4.9) | 0.6656  0.5767 |
| SCOPA-Sleep DS | 3.3 (2.6) ^ad**^ | 2.4 (2.1) ^bd***^ | 3.6 (3.1) ^cd***^ | 8.9 (4.9) | **12.25**  **<0.0001** |
| UPDRS-III | 3.1 (4) ^ac***, ad***^ | 8.8 (8.5) ^bc***, bd***^ | 24.2 (10.2) ^ac*** bc***, cd**^ | 36.7 (15.3) ^ad*** bd***, cd**^ | **32.816**  **<0.001** |
| DDE (mg) | 0 (0) ^ac**^ | 0 (0) ^ac**^ | 359.4 (262.2) ^ac** bc**^ | 238.2 (509.1) | **6.479**  **<0.001** |
| Cholinergic (mg) | 0 (0) | 0 (0) | 0 (0) | 6.0 (4.3) | **32.6**  **<0.001** |

**Table S1 Average and standard deviation of demographic, clinical and sleep measures.**

Data presented as mean (standard deviation). F, female. MoCA, Montreal Cognitive Assessment. MMSE, Mini-Mental State Exam. HADS-A, Hospital Anxiety and Depression Scale-Anxiety. HADS-D, Hospital Anxiety and Depression Scale-Depression. MEQ, Morningness-Eveningness Questionnaire; ESS, Epworth Sleepiness Scale. SCOPA-Sleep (NS), Scales for Outcomes in Parkinson’s Disease-Sleep (Nocturnal Sleep). SCOPA-Sleep (DS), Scales for Outcomes in Parkinson’s Disease (Sleep-Daytime Sleep). Bolded values denote significant difference in ANOVA (* *P*<0.05; ** *P*<0.01, *** *P*<0.001; **a** represents HC, **b** iRBD, **c** PD and **d** DLB in the Tukey’s post-hoc analysis).

**Table S2.** Cosinor analysis of actigraphy, melatonin and *Bmal1* expression.

| Cosinor analysis | HC | iRBD | PD | DLB | *P* values |
| --- | --- | --- | --- | --- | --- |
| Activity Mesor | 98.9 (18.7)  [98.5 (15.4)] | 82.1 (27.5)  [78.7(28.5)] | 67.8 (34.4)  [52.6(52.0)] | 57.8 (26.4)  [52.2(36.4)] | P < 0.001 |
| Activity Amplitude | 87.4 (13)  [86.8 (6.1)] | 69.2 (27.1)  [64.4(23.2)] | 56.3 (27.3)  [47.6(35.8)] | 47.2 (27.3)  [40.9(23.8)] | P < 0.001 |
| Activity Acrophase | 14.1 (1.9)  [13.5(2.4)] | 14.5 (1.3)  [14.7(1.4)] | 13.8 (0.9)  [13.9(1.2)] | 13.5 (0.7)  [13.4(0.5)] | NS |
| Melatonin Mesor | 3.1 (1.8)  [2.4(2.6)] | 3.01 (2.1)  [2.7(3.1)] | 3.33 (2.4)  [2.8(2.6)] | 3.01 (1.9)  [2.7(2.6)] | NS |
| Melatonin Amplitude | 3.2 (2.4)  [2.4(2.2)] | 2.7 (2.4)  [2.6(3.2)] | 2.5(2.3)  [1.2(3.3)] | 3.0 (3.0)  [1.8(2.5)] | NS |
| Melatonin Acrophase | 2.2 (2.1)  [1.8(2.9)] | 3.4 (4.1)  [2.9(3.8)] | 2.5 (2.06) [2.7(2.3] | 7.4 (5.1)  [5.9(9.7)] | NS |
| *Bmal1* Mesor | 38.4 (7.8)  [39.2(11.4)] | 43.1 (15.5)  [38.2(24.9)] | 43.8 (15.5)  [41.9 (19.4)] | 42.7(13.9)  [44.6(22.4)] | NS |
| *Bmal1* Amplitude | 28.1 (6.2)  [27.9(7.2)] | 23.1 (11.4)  [22.9(14.8)] | 23.1 (9.4)  [21.1 (11.1)] | 20.4 (4.5)  [19.9(5.1)] | P < 0.05 |
| *Bmal1* Acrophase | 1.9 (1.8)  [2.4(2.7)] | 1.2(3.6)  [1.5(3.09)] | 1.3 (4.5)  [0.8(4.9)] | 24.1 (4.7)  [23.88(3.2)] | NS |

Average for all variables with standard deviation in between brackets is given for all variables. Median and interquartile range in brackets is given in square brackets. Rhythmicity differences between groups were assessed using the Jonckheere-Terpstra test for ordered alternatives. The level of statistical significance was set at *P*<0.05.

**Table S3. Average and standard deviation of actigraphy variables.**

| Sleep variable | HC | iRBD | PD | DLB | ANOVA F  p value |
| --- | --- | --- | --- | --- | --- |
| Bedtime | 22:54 (1:17) | 23:01 (0:41) | 22:33 (0:56) | 22:18 (0:32) | 2.029  0.125 |
| Risetime | 7:33 (1:04) | 7:27 (01:12) | 6:50 (0:43) | 7:45 (0:42) | 1.183  0.328 |
| Average Time in bed (min) | 519.4 (77.2) | 505.5 (75.8) | 497.1 (53.3) | 567.1 (47.5) | 2.330  0.088 |
| Total Sleep Time (min) | 447.1 (80.5) | 440.7 (64.2) | 447.9 (45.9) | 459.3 (68.5) | 0.188  0.904 |
| Wake after Sleep Onset (min) | 45.8 (16.6) | 44.7 (23.9) | 24.5 (8.7)^cd**^ | 73.4 (40.4) | **5.273**  **0.004** |
| Fragmentation % | 27.9 (6.8) ^ad*^ | 30.9 (10.1) | 19.7 (6.6) ^cd***^ | 44.7 (16.8) | **6.008**  **0.002** |
| Sleep time in 24 hours (min) | 654.2 (117.6) | 701.3 (101.5) | 754.8 (140.1) | 781.6 (119.6) | 1.963  0.135 |
| Activity counts 24 hours | 268616 (49589.8) ^ac*^ | 219963.9 (87545.4) | 184697.9 (93230.9) | 146191.4 (66487.7) | **4.478**  **0.008** |
| Sleep efficiency % | 85.8 (5.5) | 85.4 (9.9) | 90.3 (4.8) | 80.9 (10.2) | 1.566  0.212 |
| Activity counts during night | 10080.4 (2907.9) | 9065.5 (4385) ^bd*^ | 5275.8 (1474.8) ^cd***^ | 15915 (9385.2) | **3.739**  **0.018** |
| %Wake during night | 10.3 (3.7) | 9.5 (4.8) | 6.3 (2.5) ^cd**^ | 14.2 (7.9) | **5.777**  **0.002** |
| %Wake during day active | 79.3 (7.8) | 71.3 (14.1) | 67.4 (14.1) | 63.5 (11.5) | 2.705  0.058 |

Bolded values denote significant difference in the Tukey Post hoc analyses (**P*<0.05; ** *P*<0.01; ****P*<0.001; **a** represents HC, **b** iRBD, **c** PD and **d** DLB).
